# Phytoscreening the homes of pregnant residents for volatile organic compounds: a pilot study in metro Detroit

**DOI:** 10.3389/fenvh.2026.1784584

**Published:** 2026-06-01

**Authors:** Brendan F. O’Leary, Jennifer K. Straughen, Andrea E. Cassidy-Bushrow, Connor Socrates, Mei Lu, Qiong Zhang, Shirley A. Papuga, Glen Ray Hood

**Affiliations:** 1 Department of Biological Sciences, Wayne State University, Detroit, MI, United States,; 2 Center for Leadership in Environmental Awareness and Research (CLEAR), Wayne State University, Detroit, MI, United States,; 3 Department of Public Health Sciences, Henry Ford Hospital, Detroit, MI, United States,; 4 Henry Ford Health + Michigan State University Health Sciences, Detroit, MI, United States,; 5 Department of Obstetrics, Gynecology and Reproductive Biology, College of Human Medicine, Michigan State University, East Lansing, MI, United States,; 6 Department of Pediatrics and Human Development, College of Human Medicine, Michigan State University, East Lansing, MI, United States,; 7 Department of Environmental Science and Geology, Wayne State University, Detroit, MI, United States

**Keywords:** brownfields, BTEX, preterm birth, risk-based screening level (RBSL), tetrachloroethylene (PCE), trichloroethylene (TCE)

## Abstract

Exposure to anthropogenic volatile organic compounds (VOCs) has been associated with preterm birth, yet the spatial distribution of VOC exposures within urban environments remains poorly characterized, particularly for vapor intrusion pathways. Plants have recently been used as a cost-effective, non-invasive tool to “phytoscreen” for belowground contaminants. Here, we evaluated the use of phytoscreening within a birth cohort in metro Detroit, Michigan, USA by (a) assessing participant willingness to allow sampling in residential front yards, (b) quantifying VOC concentrations across plant tissue types (leaves and twigs), and (c) testing whether VOC detections in plants are associated with proximity to brownfield sites. Of 20 pregnant participants, 18 agreed to phytoscreening and sampling was completed at 13 residences, indicating high feasibility. Across 15 plant species, five of six target VOCs (benzene, toluene, ethylbenzene, xylene, trichloroethylene, tetrachloroethylene) were detected. Specifically, toluene was detected at all 13 homes and in 68% of samples. While less common, ethylbenzene was detected in 12 samples across 3 homes and trichloroethylene was detected in one sample that exceeded Michigan’s risk-based screening level for soil vapor intrusion. Additionally, VOCs were detected more frequently and at higher concentrations in leaves compared to twigs. However, VOC concentrations in plants were not associated with proximity to known or suspected brownfield sites in this small sample size. Overall, these findings demonstrate the feasibility of phytoscreening in epidemiological studies and highlight its utility for characterizing spatial patterns of VOC exposure. Incorporating plant-based sampling approaches may help identify localized hotspots of human exposure and inform targeted environmental remediation efforts.

## Introduction

Urban communities grapple with diverse environmental challenges, with one of the most prominent being the negative impacts of environmental contamination on human health (1, 2). One common group of urban contaminants are anthropogenic volatile organic compounds (VOCs), a large group of chemicals originating from manufactured products that vaporize readily at room temperature (3). In 2020, Cassidy-Bushrow et al. (4) found an association between airshed VOCs, specifically benzene, toluene, ethylbenzene, and xylene (collectively referred to as BTEX), and preterm birth in Detroit, Michigan, USA. The city of Detroit has one of the highest preterm birth rates among large cities in the United States (5) and is home to >4,000 brownfield sites which are complicated by either potential or known presence of environmental contamination (6). Moreover, VOCs originating from subsurface release from leaking underground storage tanks have been documented at approximately 3,300 of these locations (7). It is therefore critical to develop rapid, sensitive and cost-effective screening methods to determine “hotspots” of contamination. Ultimately, our goal is to couple phytoscreening results with geospatial statistical techniques to provide a novel approach to identify areas to focus remediation efforts that results in the largest positive impact on human health (8, 9).

However, measuring belowground VOCs is difficult due to their multiphase nature, i.e., they can be adsorbed to the soil, dissolved in the aqueous phase in pore spaces, and/or dissolved in the air of the pore spaces themselves (10–13). Recently, researchers have turned to plant-based “phytoscreening” methods that analyze aboveground plant tissue to determine the presence or infer the absence of contaminants originating belowground in the rhizosphere, the region of the soil in the vicinity of roots (13–15). Unlike conventional methods such as sampling soil and groundwater that target only one of these phases, plants use their roots to uptake oxygen, water and VOCs partially dissolved therein from the subsurface where they are transported to the aboveground portion of the plant from each of the aforementioned phases (16). Consequently, phytoscreening has emerged as a cost-effective, non-invasive alternative to conventional contaminant screening methods that can reduce sampling time and increase access to private property to assess site conditions (17, 18). As a result, phytoscreening has been used in a number of scenarios related to environmental assessment and human health, for example to optimize groundwater well placement (17), map the subsurface distribution and concentrations of pollutants (19) and assess the risk of exposure (9, 20).

In the current study, we extend phytoscreening beyond simply characterizing the source and scope of an underground plume and test the feasibility of incorporating this method into an epidemiological study. Specifically, we explored the use of phytoscreening as a feasible alternative to conventional methods to assess the potential for residential exposure at the homes of pregnant residents in metro Detroit. In urban areas, especially those in cities such as Detroit with aging infrastructure (3), vapor intrusion via the upward migration of VOCs from groundwater through cracks in foundations and openings such as pipes or sumps in basements or crawl spaces (21) is considered the pathway with the greatest potential to result in human exposure (22). This pathway is also included in the Hazard Ranking System that the U.S. Environmental Protection Agency uses to place uncontrolled waste sites on the National Priorities List (23). While phytoscreening does not directly measure vapor intrusion into a home, plants have been used as an effective screening tool for initial assessment by detecting the presence or inferring the absence of VOCs at a property (9, 20, 24). Moreover, unlike conventional methods such as soil or groundwater sampling, phytoscreening is less invasive for property owners (25), which may enhance participation among populations historically hesitant to engage in health-related surveillance efforts (26).

The objectives of this study were three-fold: (a) test the feasibility and acceptability of implementing phytoscreening to survey for VOCs in the front yards of the homes of pregnant residents in metro Detroit, (b) compare the concentration of VOCs between different plant tissues types (leaves and twigs) and (c) determine if VOCs present in plants at the homes of pregnant participants is associated with proximity to brownfield locations. To our knowledge, this initial assessment study represents the first case of combining phytoscreening within the framework of an epidemiological study, with the ultimate goal of benefitting human health by informing increased monitoring and cleanup efforts in targeted areas where populations are particularly vulnerable to VOC exposure.

## Materials and methods

### Study cohort

From September 9, 2020, through July 20, 2021, pregnant persons who were patients at Henry Ford Health (HFH), which provides medical care to 20%–30% of the metropolitan Detroit population, were recruited to establish a birth cohort study to examine environmental influences on preterm birth risk. The potential participants, identified using the HFH electronic medical record (EMR) system, included those with estimated due dates from September 2020 through August 2021, receiving prenatal care at any HFH Women’s Health Clinic, ages 18–45 with a singleton pregnancy and who had a residential address at the time of prenatal care initiation within the city of Detroit. Those patients unable to read or speak English were ineligible to participate in the study.

The eligible pregnant persons were emailed letters introducing the birth cohort study with an invitation to participate via a link that allowed them to join and digitally sign the online electronic informed consent. Those HFH patients that did not respond to the link were contacted by trained interviewers via telephone. Additionally, pregnant persons without a valid email in the EMR system were contacted by phone to determine if they were interested in participating in the study at which time they were asked to provide a valid email address to receive the study link. A total of 1,085 potentially eligible pregnant patients were identified and 72 (6.6%) enrolled in the overall birth cohort study. An electronic informed consent with electronic signature was obtained for all participants and Health Insurance Portability and Accountability Act (HIPAA) authorization was obtained for a subset of birth cohort participants. This study was conducted in accordance with the local legislation and institutional requirements.

As part of the electronic informed consent process, these birth cohort participants were invited to join an optional sub-study to sample and screen plants from their front yards for VOCs common in post-industrial cities. The participants opting into the sub-study were notified of the date and time that we would be obtaining the plant samples, but they were not required to be home for phytoscreening. The studies involving human participants were reviewed and approved by the Henry Ford Health Institutional Review Board (IRB #13306–01).

### Sample collection

**FIGURE 1:**
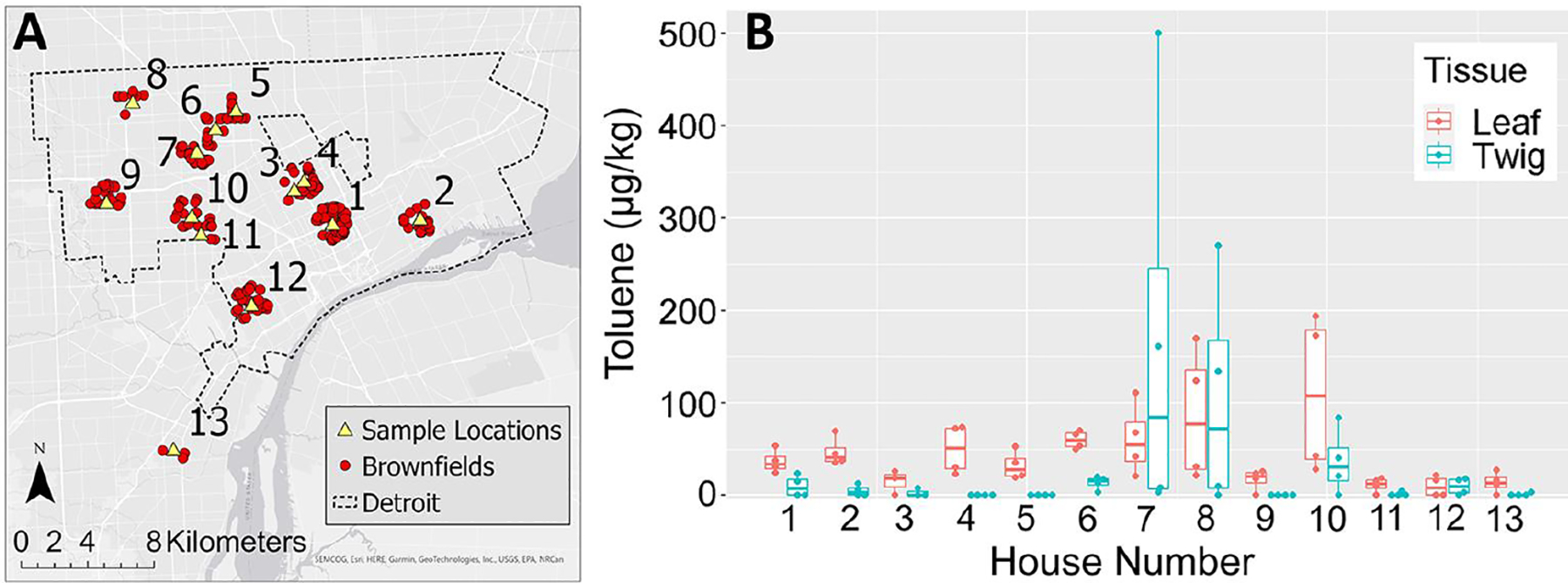
**(A)** Map of the location of 13 homes phytoscreened in metro Detroit (yellow triangles) and the brownfields located within 1-km of each home (red dots). **(B)** Boxplot of the concentrations of toluene, the most common VOC observed in the study, detected from phytoscreening leaves (red) and twig (blue) from the front yards of homes of 13 pregnant participants in metro Detroit.

At the outset of the growing season on October 2, 2020, we sampled plants from the front yards of pregnant residents (Figure 1A). This sampling period occurred within the growing season but prior to the first frost when plants go dormant in southeast Michigan, which typically occurs in mid-October (27). Traditionally, phytoscreening is performed by boring a thin cylinder of wood, known as a core, from the trunk of a tree. Unfortunately, core boring can negatively affect tree health and can be considered invasive given that many arborists recommend that no more than one or a few cores be sampled from an individual adult plant during its life while it is ill-advised to core immature trees (28–30). Additionally, trees may not be available for phytoscreening at all locations (17), especially in urban areas (31). A number of previous studies also show that sampling other tissues such as leaves and twigs from plants other than trees can detect volatile and semi-volatile compounds in similar concentrations as tree cores (18, 24, 28). Taking these considerations into account, and to minimize residential disturbance while enabling comparison across homes, we sampled and compared the concentration of VOCs in leaves and twigs (new shoot growth) from trees (when available) and shrubs and vines, which were common at all locations.

In accordance with standard environmental site assessment practices, sampling two locations in the front yards is generally considered acceptable to characterize conditions for an initial screening given the typical size of Detroit residential lots of approximately 975 m^2^ (32–34). Following this spatial sampling protocol and established plant tissue sampling methods outlined in Hood et al. (18) and O’Leary et al. (24), we sampled three to four replicates of both leaves and twigs from plants at two different locations in the front yard that were chosen because they were growing accessibly within several meters of the home’s foundation, prioritizing proximity to the building where vapor intrusion is more likely to occur, while also minimizing disturbance to landscaped areas. For each replicate from each plant at each home, five leaves or three to four twigs measuring ~10–15 cm in length were cut from the plant. This tissue was homogenized with gardening shears and ~10 g were placed in a 40-mL amber glass to meet the mass requirements for chemical analysis. The vial was then filled with 20 mL of purge and trap methanol and capped with a silicone septum. Each sample vial was immediately placed on ice, transported to laboratory, and cold-stored until chemical analysis.

### Chemical analysis

The samples were analyzed via gas chromatograph/mass-spectrometry (GC/MS) methods outlined in Hood et al. (18) for the following six VOCs commonly found in post-industrial urban areas, and previously documented in Detroit and the surrounding metro area (8, 35): benzene, toluene, ethylbenzene, total xylene (BTEX), tetrachloroethylene (PCE), and trichloroethylene (TCE). For all statistical analyses, samples below the method detection limit were coded as one-half the detection limit (Supplementary Table S1) (14, 36, 37). The concentrations of each of the six VOCs in each plant sample were compared, for context only, to Michigan’s Department of Environment, Great Lakes and Energy’s (EGLE) risk-based screening levels (RBSL) for vapor intrusion, a measurement below which VOC concentration detected in soil mass and pore water are considered protective of human health (38). While plant-specific risk bases screening levels do not exist, we focused on comparing soil RBSLs, pore water concentration per unit mass of soil (μg/kg) were evaluated alongside VOC concentrations measured in plant tissues, which were expressed as xylem water concentration per unit dry mass of plant tissue (μg/kg). This approach maintains consistency in units based on dry mass and water content, unlike comparisons involving soil vapor or groundwater, which represent concentrations in vapor or aqueous phases without reference to solid mass. Moreover, previous studies have shown the concentration of VOCs in plants and soil are often correlated (11, 16).

To determine if the concentration of VOCs phytoscreened at homes was related to their proximity to known or potential sites of contamination (brownfields), the number of brownfields within 1-km of each home (Figure 1A) was extracted from EGLE’s online database (71) and calculated using the geospatial methods of O’Leary and Miller (39). A 1-km search distance was selected to account for the potential for subsurface vapor migration and off-site impacts that can extend well beyond individual property boundaries, particularly in heterogeneous and disturbed urban settings (40, 41). While 1-km may seem like a course measurement, prior studies have evaluated these relationships across spatial scales ranging from distances of 1 km up to 10 km (42–44). The resulting values were used as the dependent variable in the correlation analyses described below (Supplementary Table S2).

### Statistical analysis

We summarized and compared the demographic characteristics of the pregnant participants in the phytoscreening study to the general pregnant population in Detroit in 2019 (45). Given that toluene was the only VOC detected in the majority of samples, we compared the concentration of toluene in leaves and twigs using a non-parametric Wilcoxon signed-rank test due to the non-normal distribution of the data. To determine if the concentration of VOCs detected were associated with the density of brownfields within 1-km of our study sites, we calculated Spearman’s rank correlation coefficients using the average concentrations of both toluene (pooled across leaf and twig samples at each home) and average total VOCs (the sum of the average concentration of each VOC detected at each home). All statistical analyses were performed in R ver. 4.4.3.

## Results

### Demographics of the participants

In total, 20 pregnant participants enrolled in the birth cohort study during the plant growing season, with 18 (90%) agreeing to have plants in their front yard sampled and phytoscreened. However, despite obtaining property access agreements and establishing a sampling timeframe, plant samples were ultimately collected from only 13 homes due to logistical constraints including field team availability, resident scheduling conflicts and a limited sampling window prior to first frost. Of these, eight participants signed a HIPAA authorization form, which allowed linkage of their demographic data with the phytoscreening results. The demographics of these participants were similar to that of the pregnant population in Detroit in 2019 with notable exceptions. For example, while the average age of the 8 pregnant participants with HIPAA authorization was 27.5 ± 5.8 years, the proportion of those under 20 years of age was slightly higher (12.5%) compared to Detroit (8.3%). Moreover, participants in the phytoscreening study were more educated; five participants (62.5%) had at least some college education, and all participants had at least a high school education compared to 79.6% of pregnant persons in Detroit. While most births in Detroit were to Black pregnant persons (80.5%), in the phytoscreening study, 62.5% of the participants were Black (36, 46). Moreover, study participants were more likely to be married (25%) compared to the pregnant population of Detroit in 2019 (20.4%).

### Phytoscreening for VOCs

A total of 102 plant samples from leaves (*n* = 51) and twigs (*n* = 51) were collected from the front yards of 13 homes from a total of 15 different plant species (Table 1). While five of the six VOCs were detected in at least one leaf or twig sample, only toluene was detected at all 13 homes (Figure 1B, Table 2) in 68% of the samples (*n* = 69). The second most common VOC, ethylbenzene, was detected in 12 samples at three homes at an average concentration of 379 (±28.4) μg/kg, well above EGLE’s soil risk-based screening level (RBSL) of 12 μg/kg for vapor intrusion in all instances. Similarly, TCE was detected at a concentration of 8.8 μg/kg at a single home, exceeding soil RBSL of 0.33 μg/kg (Table 2). In contrast, PCE was detected at two homes, and TCE at a single home, while benzene was not detected. While ethylbenzene was detected in both leaves and twigs (Supplementary Table S1), PCE and TCE were detected in only a single twig at one home and a single leaf at two homes, respectively. The concentration of toluene was significantly higher in leaves (42.7 ± 6.06 μg/kg) than in twigs (28.0 ± 11.5 μg/kg) (*P* = 0.01) (Figure 1B). Moreover, fewer leaves (12%) than twigs (53%) were below the detection limit for toluene. There was no association detected between either the concentration of toluene (*r*_*s*_ = 0.11; *P* = 0.71) or total VOCs (*r*_*s*_ = −0.04; *P* = 0.90) and the density of brownfields within 1-km of the 13 homes that were phytoscreened in this small sample size.

## Discussion

Our study suggests that pregnant research participants in Detroit were agreeable to having phytoscreening conducted at their residence and that plants can detect VOCs in the front yards of homes. Importantly, several different plant species detected five of six VOCs, with toluene detected at all 13 homes and leaves detecting higher concentrations of VOCs at a greater frequency above the method detection limit compared to twigs. While the presence of VOCs is not associated with proximity to known or potential sources of VOC contamination with this limited sample size, we did identify three homes where a total of 13 samples exceeded Michigan’s soil RBSL for ethylbenzene and TCE. While detection above EGLE’s RBSL raises concerns, our results underscore the value of phytoscreening as a pragmatic tool for assessing residential properties for potential VOC issues. Given these results, our ultimate goal is to use plants to locate VOC “hotspots” in residential areas across Detroit to target for remediation. In fact, the simplicity of collecting samples for phytoscreening may enable property owners interested in participating in research efforts to collect and send samples to testing facilities to protect their own well-being by aiding in public health screening and reporting efforts.

An interesting dichotomy exists in our VOC data: toluene was present at every home and in the majority of samples, while benzene was not detected despite being common in Detroit and other large industrial US cities as well as other phytoscreening studies (15, 24, 47). Interestingly, most brownfields included in our geospatial analysis harbor leaking underground storage tanks, which are often sources of both benzene and toluene. This begs the questions, why do we see such disparity in the detection of benzene vs. toluene and why is there a lack of a relationship between VOC concentrations and proximity to brownfields? First, benzene is more volatile than toluene, potentially exhibiting a greater potential to migrate through the soil matrix (48) or move quickly through the plants itself (49, 50). However, toluene is known to penetrate plant cuticles (51) and has been documented to accumulate in plants for longer periods of time (52, 53). Second, we acknowledge that small sample size limited the scope and statistical power of our geospatial analyses, with most brownfields located over 500 m from the studied homes, well beyond the 10- to 30-m lateral inclusion zone that EGLE defines for VOCs. Under conventional conceptual models, highly volatile compounds such as benzene are not expected to migrate from distant subsurface sources into residential areas prior to volatilization (54), particularly beyond regulatory inclusion zones. However, recent vapor intrusion studies have identified conduit-mediated transport as a significant pathway, with vapor migration distances exceeding 150 m (55, 56). This spatial mismatch represents a conceptual limitation of the geospatial analysis, indicating misalignment with conventional vapor intrusion models and underscoring the need to evaluate alternative transport mechanisms across multiple spatial scales. At present, we are expanding our phytoscreening efforts to include sites closer to brownfields to increase our sample size and allow for a more robust statistical test of this hypothesis.

Plant sampling was conducted on a single day and did not explicitly assess seasonal effects on VOC concentrations, which represents a temporal limitation of our study. However, investigating variation in the plant tissue concentrations detected across the field season is an important consideration for future work. Variation in the capabilities of plants to detect VOCs across the growing season have been observed in previous phytoscreening studies (30), but these patterns are often contrary to expectations. For example, in more northern climates like Detroit, plants typically uptake large quantities of nutrients and water during the growing season (spring and summer) and reduce their intake in the fall as they slow their metabolism to transition to winter dormancy (17). Yet contaminant uptake may not always follow this seasonal trend and instead may vary due to site-specific conditions. External factors, such as groundwater levels, may play a significant role in driving plant VOC uptake. In Detroit, for instance, the water table tends to rise during the winter months and lower during the summer (57), suggesting that hydrologic dynamics may play a more important role in contaminant availability than seasonal plant physiology. Additionally, phytoscreening does not directly measure vapor intrusion into indoor environments but serves as an exploratory screening tool to identify the presence or infer the absence of VOCs at a property. Plant-based detections should be interpreted alongside variability and background noise in indoor air concentrations, as well as the seasonal dynamics of vapor intrusion, which can influence indoor concentrations over time. Therefore, future studies should evaluate the combined influence of seasonality, hydrology, and other environmental variables to better understand temporal variation in phytoscreening outcomes.

In our study, we sampled both leaves and twigs because they are easy to collect, do not damage the tree and prior studies show that these tissues detect VOCs in similar concentrations to tree cores (13, 17). Additionally, collecting both tissues allowed us to compare the concentrations of VOCs between leaves and twigs. Leaves are sinks during periods of growth and metabolic activity, receiving inorganic compounds dissolved in water transported from the roots via xylem tissue required for photosynthesis (58). Our dataset therefore allowed us to test the hypothesis that leaves as sinks accumulate and therefore detect higher concentrations of VOCs than twigs. We found that leaves detected toluene in a ~1.5 times higher concentration, on average, compared to twigs and that sampling leaves produced fewer samples below detection limit. While future studies should focus on comparing multiple tissue types including leaves, twigs, tree cores and other tissues that are sinks such as flowers and fruit (59, 60), our study suggests that leaves may produce fewer false negatives and VOCs may be less transient in leaves than other tissue types.

While the sample size of the initial phytoscreening cohort is relatively small, this assessment study demonstrated the willingness of pregnant participants in Detroit. This point is critical; from the perspective of researchers, what might seem like a “simple snip of a twig in the name of human health sciences” may be more complicated for the Detroit community. Participation in research efforts in cities like Detroit may harken back to years of historically misaligned goals and injustices that have fostered mistrust and distrust of medical and academic researchers and politicians alike (61, 62). We acknowledge that qualitative data on community perceptions or barriers to participation were not collected but can be incorporated into the study in the future. Additionally, while the recruitment rate into the overall epidemiological cohort (~6.7%) is higher than other recent birth cohort studies in urban areas [e.g., ~2.5% reported in Wilhite et al. (63)], we recognize that this limits generalizability of the study findings and that selection bias may be present. Although maintaining and increasing participation in these cohorts presents challenges (64), future studies will aim to include a larger, more geographically representative cohort and incorporate measures of individual exposures and associated health outcomes. Engaging research participants in the collection process and reporting back of individual results should also be considered to increase engagement in study activities and to create greater trust.

## Conclusions and future directions

In this initial assessment study, we showed that phytoscreening provides a useful, non-invasive starting point to determine potential exposure to VOCs. Such screening methods are valuable for assessing the effects of exposure on human health as well as identifying sites for potential remediation. However, while there are soil vapor intrusion RBSL, there are currently no equivalent RBSL for plants. Development of a plant-based RBSL is clearly needed prior to adopting phytoscreening in lieu of more expensive and labor-intensive conventional methods of screening well water or soil for VOC contamination that required personnel with specialized training. Moreover, a lack of a plant-based RBSL is particularly problematic given that Detroit has more urban gardens, per capita, than any other major US city, and urban agriculture is increasingly becoming an important way in which people gain access to fresh produce (65, 66). Lastly, plants produce their own suite of VOCs used to attract pollinators (67), deter herbivores (68), and communicate with neighboring plants (69, 70). Of the six anthropogenic VOCs we screened in our study, toluene, ethylbenzene and xylene are the only compound that are naturally produced in small amounts as part of their natural metabolic process (52, 53), albeit typically below detection limits of modern laboratory methods. While it seems unlikely these VOCs are produced in high concentrations by 15 different plants species from 14 different plant families, it remains unknown how taxonomically and seasonally widespread the natural production of plant-based toluene, ethylbenzene and xylene. In the future, we will develop methods to differentiate between plant vs. anthropogenic VOCs.

In conclusion, by demonstrating (a) a high response rate for implementing phytoscreening at the residences of pregnant participants and (b) that detection rates and elevated concentration values observed in leaf samples compared to twigs, this initial assessment forms the basis for a larger cohort study. While our cohort was adequately spatially distributed, increasing the density of sampling sites in future phases of this research will allow more statistical power to evaluate the relationship between brownfield proximity, density, and residential VOC levels in Detroit. Importantly, these findings highlight the need to address the ongoing issues associated with unscreened brownfield sites in urban centers across the United States to better understand the redevelopment potential of commercial properties but also emphasize the importance of understanding residential home exposures near these sites of potential contamination. Ultimately, our results lay the groundwork for integrating phytoscreening into broader vapor intrusion assessments and remediation strategies, supporting efforts to reduce VOC exposures and protect vulnerable populations.

## Supplementary Material

Supplemental Material

The Supplementary Material for this article can be found online at: https://www.frontiersin.org/articles/10.3389/fenvh.2026.1784584/full#supplementary-material

## Figures and Tables

**TABLE 1:** Common name, family, genus and species of the plants sampled at the 13 residences of pregnant participants in metro Detroit.

Home number	Common name	Family	Genus and species	Average concentration (and range) of toluene
1	Ornamental cherry	Rosaceae	*Prunus* sp.	23.2 (0.86–53.2)
	Mulberry	Moraceae	*Morus* sp.	
2	Rose	Rosaceae	*Rosa* sp.	26.3 (2.58–69.5)
	Tree of Heaven	Simaroubaceae	*Allanthus altissima*	
3	Rose of Sharon	Malvaceae	*Hibiscus syriacus*	8.98 (0.86–25.8)
	Mulberry	Moraceae	*Morus* sp.	
4	Dogwood	Cornaceae	*Cornus* sp.	25.3 (0.86–73.9)
	Ninebark	Rosaceae	*Physocarpus opulifolius*	
5	Dogwood	Cornaceae	*Cornus* sp.	16.5 (0.86–52.5)
	Grapevine	Vitaceae	*Vitus riparia*	
6	Oleander	Apocynaceae	*Nerium oleander*	36.6 (3.53–70.1)
7	White cedar	Cupressaceae	*Thuja occidentalis*	114.2 (3.27–500)
	Mulberry	Moraceae	*Morus* sp.	
8	White cedar	Cupressaceae	*Thuja occidentalis*	95.1 (0.86–270)
	Mulberry	Moraceae	*Morus* sp.	
9	Grapevine	Vitaceae	*Vitus riparia*	9.10 (0.86–25.9)
	Black walnut	Juglandaceae	*Juglans nigra*	
10	White cedar	Cupressaceae	*Thuja occidentalis*	72.9 (0.86–194)
	Mulberry	Moraceae	*Morus* sp.	
11	Ornamental Paper birch	Betulaceae	*Betula papyrifera*	6.63 (0.86–18.8)
	Burning bush	Celastraceae	*Euonymus alatus*	
12	Silver maple	Sapindaceae	*Acer saccharinum*	9.66 (0.86–21.4)
13	Redbud	Fabaceae	*Ceris canadensis*	7.78 (0.86–27.1)
	Ornamental cherry	Rosaceae	*Prunus* sp.	

In most cases, this is the first documented case of these plant species being sampled and used in a phytoscreening study. At homes 6 and 12, where a single plant is listed, two individuals of the same plant species were phytoscreened. Not all plants were identifiable to the species level. Also given are the average and range of concentrations of toluene (μg/kg), the only VOC detected from each of the 13 homes (Figure 1B). The location of each home is given in Figure 1A.

**TABLE 2:** The six volatile organic compounds (VOCs) phytoscreened in this study at 13 homes in metro Detroit, including the number of homes and plant samples from which they were detected, Michigan’s department of environment, great lakes, and energy risk based screening level (RBSL) for soil vapor intrusion [32], and their average concentration (pooled across leaf and twig samples) and standard deviation (±SD).

VOCs phytoscreened	Number homes Detected (*n* = 13)	Leaf samples detected (*n* = 51)	Twig samples detected (*n* = 51)	Soil vapor RBSL (μg/kg)	Number samples above RBSL (*n* = 102)	Average concentration ± SD (μg/kg)
Benzene	0 (0%)	0 (0%)	0 (0%)	1.7	0 (0%)	ND
Toluene	13 (100%)	45 (88%)	24 (47%)	3,700	0 (0%)	51.7 ± 74.9
Ethylbenzene	3 (23%)	6 (12%)	6 (12%)	12	12 (12%)	**379 ± 28.4**
Total xylene	2 (15%)	4 (8%)	0 (0%)	280	0 (0%)	173 ± 206
Tetrachloroethylene	2 (15%)	2 (4%)	0 (0%)	6.2	0 (0%)	2.71 ± 0.140
Trichloroethylene	1 (7%)	0 (0%)	1 (2%)	0.33	1 (1%)	**8.87**

The percentage of homes as well as leaf and twig samples from which each VOC was detected are given in parentheses. Averages above the RBSL are bolded.

## Data Availability

The raw data supporting the conclusions of this article are included in the supplementary material, further inquiries can be directed to the corresponding author/s.
